# COSUMO: study protocol for the development of a core outcome set for efficacy and effectiveness trials in posterior segment-involving uveitis

**DOI:** 10.1186/s13063-017-2294-8

**Published:** 2017-12-01

**Authors:** Mohammad O. Tallouzi, Jonathan M. Mathers, David J. Moore, Philip I. Murray, Nicholas Bucknall, Jane M. Blazeby, Melanie Calvert, Alastair K. Denniston

**Affiliations:** 1 Institute of Applied Health Research, College of Medical and Dental Sciences, University of Birmingham, Edgbaston, Birmingham, B15 2TT UK; 2 Academic Unit of Ophthalmology, Institute of Inflammation and Ageing, College of Medical and Dental Sciences, University of Birmingham, Birmingham, B15 2TT UK; 3 Patient Involvement Group in Uveitis (PInGU), Birmingham, B15 2TT UK; 4 Centre for Surgical Research, School of Social & Community Medicine, University of Bristol, Canynge Hall, 39 Whatley Road, Clifton, Bristol, BS8 2PS UK; 5 Centre for Patient Reported Outcome Research, Institute of Applied Health Research, College of Medical and Dental Sciences, University of Birmingham, Edgbaston, Birmingham, B15 2TT UK; 6 Department of Ophthalmology, Queen Elizabeth Hospital Birmingham, University Hospitals Birmingham NHS Foundation Trust, Birmingham, B15 2WB UK; 7 NIHR Biomedical Research Centre at Moorefield’s Eye Hospital NHS Foundation Trust and UCL Institute of Ophthalmology, London, EC1V 2PD UK

**Keywords:** Uveitis, Core outcome set, Macular oedema, Domain, Delphi, Interviews, Focus group, Consensus method, Clinical trials, Key stakeholders

## Abstract

**Background:**

Uveitis, a group of disorders characterised by intraocular inflammation, causes 10–15% of total blindness in the developed world. The most sight-threatening uveitis affects the posterior segment of the eye (posterior-segment involving uveitis (PSIU)). Numerous different outcomes have been used in clinical trials evaluating alternative treatments for uveitis, limiting inter-trial comparison and aggregation of data. We aim to develop a core outcome set (COS) that would provide a standardised set of outcomes to be measured and reported in all effectiveness trials for PSIU.

**Methods:**

A three-phase design will be used informed by recommendations from the Core Outcome Measures in Effectiveness Trials (COMET) initiative. Phase 1: a comprehensive list of outcomes will be identified through both a systematic review of effectiveness trials of PSIU and qualitative research with stakeholders. The qualitative study will comprise focus groups with patients and their carers in parallel with one-to-one telephone interviews with health professionals and policy-makers. In the focus groups, patients will be grouped according to whether or not their uveitis is complicated by the sight-threatening condition uveitic macular oedema (UMO) since it is hypothesised that the presence of UMO may significantly impact on patient experience of PSIU. Phase 2: Delphi methodology will be used to reduce the range of potential outcomes for the core set. Up to three Delphi rounds will be used through an online survey. Participants will be asked to rate the importance of each outcome on a 9-point Likert scale where 9 is most important. Phase 3: a consensus meeting will be held with key stakeholders to discuss the Delphi results and ratify the final outcomes to be included in the COS.

**Discussion:**

The development of an agreed COS for PSIU would help ensure that outcomes which matter to key stakeholders are captured and reported in a consistent way. A COS for PSIU would allow greater comparison and aggregation of data across trials for the better evaluation of established and emerging therapies through evidence synthesis and meta-analysis to inform clinical guidelines and health policy.

**Trial registration:**

COMET. http://comet-initiative.org/studies/details/640. August 2015.

## Background

Uveitis describes a group of disorders characterised by intraocular inflammation responsible for 10–15% of total blindness in the developed world and up to 25% of blindness in the developing world [1–9]. Although uveitis may affect any age group, it peaks in the working-age population and has a disproportionately high impact in terms of years of potential vision loss [1] and need for long-term therapy with its socioeconomic impact being estimated to be at least as significant as that of diabetic retinopathy [9].

The most sight-threatening uveitis affects the more posterior structures of the eye, classified anatomically as intermediate, posterior and panuveitis [10, 11]. In clinical trials, these uveitic diseases are often grouped together as posterior segment-involving uveitis (PSIU) because of a number of shared features including their higher risk of sight-threatening complications and their requirement for systemic or local injection-based therapy. One of the most important complications in uveitis is uveitic macular oedema (UMO) which affects around one-third of patients with PSIU [1, 12–14]. UMO is a leading causes of sight loss in these patients and, due to its impact on the ‘central vision’ essential for reading, driving or recognising people’s faces, may be hypothesised to have a distinct impact on patients with PSIU.

There is currently a major unmet need in the treatment of PSIU with a paucity of high-level evidence to allow evaluation and licensing of therapies by regulatory authorities [15] and to inform treatment decisions by clinical experts and patients [16]. One of the major blocks identified in this area has been around ‘outcome measures’: the inadequacy of many of the standard outcomes used and inconsistency of the use of these outcomes between trials [15]. A systematic search of clinical trial registries noted that in 104 clinical trials of PSIU 14 different outcomes were used as a primary outcome, the most common being ‘visual acuity’ , ‘vitreous haze’ or ‘macular oedema’. Even where the same domain was used there was often variation in the way it was measured, analysed and reported [17]. This has seriously limited coherent evidence synthesis and meta-analysis in the field.

Inconsistent use and reporting of outcome measures can be addressed through the use of the core outcome set (COS). The COS is a standardised set of outcomes that have been scientifically agreed and are measured and reported in all trials for a specific clinical area [18]. The COS is not restrictive since other data can be collected, but rather ensures that certain key outcomes are always collected in a standardised way. This may profoundly enhance evidence synthesis by enabling comparison (due to the consistent collection of outcomes), reducing outcome-reporting bias (as the whole COS is reported) and improving the statistical power of any meta-analysis (more studies can be included). Development of the COS is supported by a number of initiatives such as the Core Outcome Measures in Effectiveness Trials (COMET) initiative [18] and has been endorsed by Cochrane and the World Health Organisation [19].

The development of a COS for PSIU would provide for the first time a standardised set of outcomes to be measured that has value to all stakeholders and can be used in all comparative efficacy or effectiveness trials in uveitis [20]. This has the potential to profoundly enhance evidence synthesis and reduce research waste [19, 21] with direct benefits to patients with sight-threatening uveitis.

### Aims and objectives

#### Aims

The aim of this study is to define a COS for PSIU for use in effectiveness trials in adult patients. In addition, we will evaluate any difference in priorities that arise from the presence or absence of the key sight-threatening complication, UMO.

#### Objectives

There are three specific objectives for the study:

1. To identify a comprehensive list of potential outcomes based on (a) systematic review of clinical trials in PSIU and (b) findings from key respondent focus groups and interviews (patients, carers, health professionals, health policy-makers).
2. To prioritise outcomes through a Delphi process, and to evaluate any potential impact of the presence or absence of UMO.
3. To discuss the Delphi results and finalise the COS for PSIU through a consensus meeting with the key stakeholder groups (patients, carers, clinicians, policy-makers).

## Methods

**Fig. 1:**
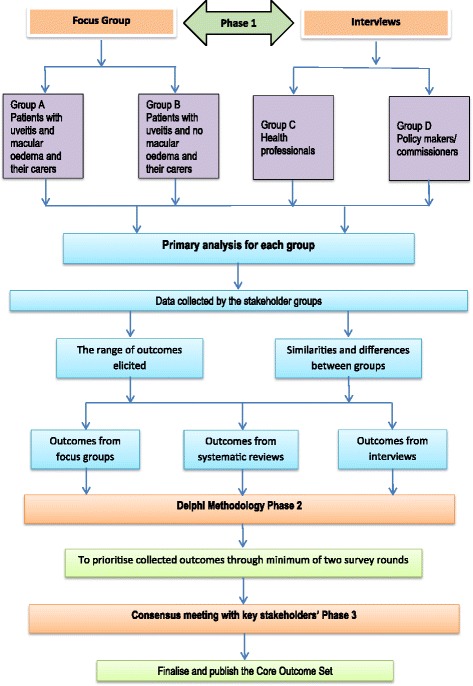
Flow diagram illustrating study design

A three-phase approach will be used to develop a comprehensive COS, in which any relevant outcome identified by any stakeholder will be considered. This may therefore include clinical parameters: patient-reported function, quality of life and health-economic factors, among others. First, a comprehensive list of outcomes will be identified through a review of outcomes reported in existing trials (systematic review) and qualitative research with stakeholders. Second, a Delphi process will be used, asking the stakeholders to prioritise outcomes for relevance through an online survey. Third, a consensus meeting will be held with key stakeholders to discuss the Delphi results and confirm the final outcomes to be included in the COS [22]. The study design is illustrated in Fig. 1.

### Phase 1: identifying a comprehensive list of potential outcomes to be included in the COS

#### Systematic reviews

A systematic review will be conducted on the effectiveness of pharmacological agents for PSIU (including PSIU with UMO) and potential outcomes will be identified for inclusion in the COS. The identified outcomes from the systematic review will be used to supplement the list of outcomes identified in the qualitative research. The combined list will be reviewed to make sure they provide clear meaning and no duplication [23].

Standard systematic review methodology will be employed to identify, select and extract data from comparative studies (randomised/non-randomised and observational studies) of pharmacological interventions in patients with UMO. Searches will be conducted through bibliographic databases (Cochrane Library, MEDLINE, EMBASE and CINAHL) and clinical trials registers. No restriction will be placed on either language or year of publication. Translation of non-English language articles will be undertaken to minimise selection bias. Data extraction will include the following: basic trial information and name, investigator names, year of study, primary outcome, secondary outcomes, method of measurement and analysis for all outcomes [23].

#### Qualitative research

Potential outcomes to be included in the COS will be identified through focus groups with patients and carers, and one-to-one telephone interviews with health professionals, health policy-makers and commissioners.

#### Focus groups with patients and carers

##### Participants

Participants are patients with PSIU and their carers. To be included as a ‘Patient Participant’, the individual must: have a confirmed diagnosis of uveitis involving the posterior segment of the eye (intermediate uveitis, posterior uveitis or panuveitis) with or without macular oedema; have active follow-up for their PSIU; be at least 18 years of age; have the capacity to read and write the English language as well as good spoken English; and have the capacity to give written consent. Patient participants will be allocated to either the PSIU with UMO group or the PSIU without UMO group based on whether they have had UMO within the last 2 years. For inclusion as a ‘Carer Participant’, a carer is defined as a person at least 18 years of age (e.g. friend, family member or spouse) who provides unpaid and informal care to the patient during his/her illness and treatment journey for uveitis and UMO. Carers will attend the same focus group as the patient they are accompanying.

Patients will not be included if: they have purely anterior uveitis; or they have other unrelated ocular co-morbidities such as age-related macular degeneration, or diabetic retinopathy that might have significant impact on their vision and experience of uveitis. The presence of uveitis complications such as glaucoma, cataract, retinal vasculitis and choroidal neovascularisation are not exclusion criteria, but will be recorded and reported; these complications are not expected to segregate with either the UMO or non-UMO group and therefore should be approximately equally distributed. Nevertheless, if it was clinically felt that any of these complications was the main cause of vision impairment, those patients would be excluded. Peak central macular thickness will be reported for all patients in which OCT has been undertaken as part of their clinical care. Most recent best corrected visual acuity will be reported in all cases.

##### Recruitment

Patients will be recruited from the NHS Uveitis Clinics at the Birmingham and Midland Eye Centre (Sandwell and West Birmingham Hospitals NHS Trust) and University Hospitals Birmingham NHS Foundation Trust. Eligible participants (patients and their carers) meeting the inclusion criteria will be identified by ophthalmologists (PIM and AKD). A recruitment pack will be distributed and include an invitation letter and a participant information sheet. Written consent will be taken on the day of the focus group. The recruitment process and the consent pathway are illustrated in Fig. 1.

##### Sampling

To ensure a maximum range of views and opinions are collected [24], patients will be purposively sampled according to the following key characteristics: patients with uveitis with vs without UMO; patients with currently active vs inactive disease; age; and gender. We will aim to recruit approximately six participants per focus group, with a total number of participants for this stage anticipated to be between 24 and 30. Initially, four focus groups will be conducted, two each of PSIU with and without UMO, although further groups may be convened depending on judgements of data saturation [25].

#### Data collection (focus groups)

Focus groups will seek to identify outcomes that are important to patients and their carers. Whilst a variety of approaches will be utilised in order to understand participant perspectives, key areas for discussion will include the experience of uveitis and the impact on patients’ and carers’ lives; their hopes and expectations for treatment and life with uveitis; as well as discussion about outcomes that they would prioritise. A topic guide will be used to facilitate the focus group discussion. The focus group discussions will be audio-recorded.

#### One-to-one telephone interviews with health professionals, policy-makers and commissioners

##### Participants

Participants will include health professionals who are involved in caring for patients with PSIU, health policy-makers and commissioners with an influence on uveitis care. The use of telephone interviews facilitates wide geographical coverage and is felt to be more practicable for these participants [26, 27].

##### Recruitment

Recruitment of health professionals, health policy-makers and health commissioners will be via UK and international clinical, research and health service networks. Potential participants will be contacted via email containing an invitation letter and study information sheet, with a single reminder 2 weeks later. For those who agree to participate, a reminder will be sent 2 days prior to the interview date.

##### Sampling

The aim is to recruit approximately 15–20 interviewees (depending on judgements of data saturation) to include clinical, policy and commissioning perspectives.

#### Data collection (interviews)

One-to-one telephone interviews will be conducted to identify outcomes that are important to clinical and policy participants. A topic guide broadly equivalent to that used in the patient and carer focus groups will be used as a basis for discussion. Interviews will be audio-recorded with oral consent obtained at the start of each interview.

#### Data analysis

Focus groups and interviews will be transcribed clean verbatim for analysis. A thematic analysis of content will be informed by the framework analytical approach [28]. Analysis will be conducted with reference to recordings, transcripts and field notes taken at the time of data collection. Following initial familiarisation, data will be coded and then indexed prior to establishing thematic frameworks. These frameworks will enable comparative analysis of outcomes identified between key groups, including evaluating whether the presence of UMO affects the outcomes identified by patients with PSIU. Data collection and analysis will run concurrently.

#### Synthesis of comprehensive outcome list

The results of the literature review and the qualitative study will be merged to form a single comprehensive list of outcomes. These will be finalised through discussion with representatives of stakeholder groups who will help ensure standardisation and appropriate phrasing for ease of understanding for all groups.

### Phase 2: Delphi methodology

An online Delphi study will be used to reduce the range of potential outcomes to a smaller core set [29]. A Delphi approach informed by work of the COMET Initiative will be used [18], including sequential rounds through which the participants’ opinions are sought and fed back anonymously. Additionally, participants are encouraged to re-evaluate their responses in the light of these new data [30]. There will be no direct contact between participants, but participants will be asked to participate in sequential questionnaires that constitute different rounds [29, 31]. A recruitment pack that includes an invitation to the study alongside participant information sheet will be sent to the eligible participants. A key part of COS philosophy is to ensure wide stakeholder engagement such that patients, carers, physicians and other health staff contribute to COS development. The Delphi technique has been and will continue to be an important data collection methodology with a wide variety of applications and uses for people who want to gather information from those who are immersed and embedded in the topic of interest and can provide real-time and real-world knowledge [22].

#### Recruitment

A broader group of participants representing the key stakeholder groups will be approached, via local and national patient groups and clinical and research networks, and invited to participate in the survey. Additionally, participants from the focus groups and interviews will also be invited to take part in the Delphi study. Clinicians will be invited through international expert groups while nurse practitioners will be invited via the International Ophthalmic Nurses Associations (IONA) and Moorefield’s Eye Hospital. Respondents will be sent a direct link to the online survey. The purpose of the Delphi process and participant information (e.g. voluntary participation, confidentiality/anonymity, right to withdraw) will be introduced at the front of the survey. Participants will provide consent by confirming the ‘required field’ button, which states “I have read the information provided, and agree to participate in this survey”.

#### Sampling

The sample size for the Delphi methodology is anticipated to be approximately 120 participants comprising 40 patients with PSIU (20 with UMO, 20 without UMO), 20 carers, 40 health professionals and 20 policy-makers. Although there is no consensus on the sample size used in Delphi methodology, the chosen sample size is based upon previous Delphi studies [29, 32].

### Delphi rounds

A minimum of two Delphi rounds, including all of the stakeholders’ groups, will be conducted. If consensus is not reached, however, further rounds will be considered until consensus is reached [22].

#### Delphi round one

In the first round of the Delphi process, an online survey will be introduced for completion by the participants. Agreement will be confirmed with those who wish to participate and a unique ID number will be provided to gain access to the Delphi survey. If participants are not able to have access to the online survey, a paper copy of the survey will be provided. If the survey is not completed within 5 working days of the initial date, a reminder will be sent. Participants will be asked to identify the key stakeholder group they belong to; health professionals and health policy-makers will also be asked to provide their professional role and years of experience.

The list of outcomes generated in Phase 1 (systematic review and qualitative studies) will be presented to the participants, who will be asked to rate their importance on a 9-point Likert scale (1 = no importance; 9 = critical). Two additional free-text boxes will be provided to respond to the following questions: “Do you think there are any other outcomes that should be measured in patients with uveitis affecting the back of the eye (posterior-segment involving uveitis)?”and “Any other comments?”.

**Fig. 2:**
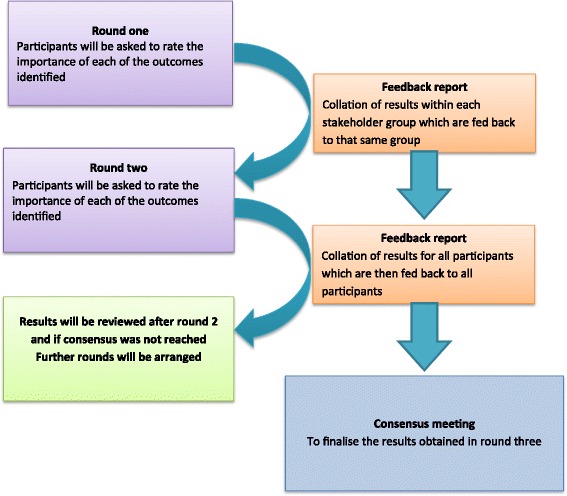
Delphi procedure. Legend: The Delphi procedure for the COSUMO study. Each round comprises an on-line questionnaire resulting in a feedback report which is presented as a supplemental file to the next round’s questionnaire. A criterion for consensus is defined in the context

At the end of round one, the response rate in each of the stakeholder groups will be assessed. If the response rate is below target, second reminders will be sent and if necessary further recruitment will be considered. If any additional outcomes have been identified by respondents these will be included in round two. The Delphi process is illustrated in Fig. 2.

#### Delphi round two

Participants from round one will be invited to participate in round two. Participants will be provided with the results from the own stakeholder group. Participants will be asked to rate the outcomes again (including any new outcomes identified in round one) on the 9-point scale. Further Delphi rounds will be conducted if consensus is not reached.

#### Delphi analysis

At the end of the final round, responses will be analysed with a view to determining whether each outcome should be included in the final COS. ‘Consensus In’ will be based on an outcome being scored 7–9 by more than 70% participants, and being scored 1–3 by fewer than 25% participants. ‘Consensus Out’ will be based on an outcome being scored 7–9 by more than 70% and being scored 1–3 by less than 25%. Outcomes for which there is no consensus will be brought for discussion to the consensus meeting.

A pre-specified secondary analysis will also be undertaken looking at the effect of stakeholder group and the impact of the presence of UMO on the scoring of outcomes.

### Phase 3: consensus meeting

The Delphi process will conclude with a face-to-face consensus meeting of key stakeholders, the aim of which is to achieve a consensus COS for PSIU. The results of the Delphi study will be fed back to participants, with greatest emphasis being placed on the results of the final round. Discussion will focus on the following three aims: approving all outcomes identified as ‘Consensus In’ at the end of the final Delphi round; considering whether further outcomes should be included when a clear rationale is provided; and to check for redundancy between outcomes. It is intended that all participants involved in Phases 1 and 2 will be invited to attend, although due to the practical constraints of a face-to-face meeting we anticipate that this will be around 20–30 people. A priority will be to ensure that there is appropriate balance of representation of the different stakeholder groups.

## Discussion

Currently there is no consensus on which outcome measures should be collected in uveitis trials. This heterogeneity of outcome renders comparison of trials and formal meta-analysis difficult or even impossible. All used outcomes currently have been largely determined by clinical experts, with minimal engagement from other stakeholders. Many of the outcomes used are not meaningful to patients, and most are not recognised as an acceptable outcome by regulatory authorities such as the Food and Drug Administration (FDA) or the European Medicines Agency (EMA). The development of a COS for PSIU would for the first time provide a standardised set of outcomes that has value to all stakeholders and can be used in all future effectiveness trials in uveitis.

The value of a COS is increasingly recognised. Benefits include maximising the value of each clinical trial since key outcomes are measured and reported in all relevant trials; ensuring that outcomes measured include those that are most important to each group of stakeholders, rather than just to one group; reducing outcome-selection bias and outcome-reporting bias since the whole COS is measured and reported; and improving the statistical power of any meta-analysis since more studies can be included [19, 20].

An important feature of most COS development is the involvement of patients and other key stakeholders from the outset, to ensure that the final COS does not simply reflect medical perceptions of the disease but includes those outcomes that matter to patients, and those which are required by regulators and policy-makers to make licensing and funding decisions. The current paradigm for outcome assessment and reporting in uveitis is based on the Standardisation of Uveitis Nomenclature (SUN) workshop in 2005. This was a major step forward in the process of standardising the methods for reporting clinical data in the field of uveitis [11, 33], but was based on the consensus of clinical experts only. Patients, carers and health professionals may differ as to which outcomes are most important, and there may be a tendency for clinicians to undervalue a number of outcomes that matter to patients [5, 34, 35]. Work in other diseases suggests that functional outcomes seem to be more meaningful to patients and may be under-represented compared to clinical and anatomic outcomes that are easier to measure [36].

Furthermore, since publication of the SUN criteria, a number of newer instrument-based measures have become available, notably optical coherence tomographic (OCT) measurement of macular thickness to detect and quantify macular oedema. Such measures provide objectivity and sensitivity to change that is attractive to researchers and triallists but may be less meaningful to patients. Our proposed COS methodology will address these issues by compiling a contemporary long list of potential outcomes derived from systematic review of trials in PSIU and additional outcomes identified by patients and other key stakeholders, which will then be prioritised through a Delphi approach and a consensus meeting to produce the final COS [37–41].

A potential influence on the output from the Delphi is the cut-off values for determining consensus. We have sought to reduce bias by stipulating these a priori. These cut-off values are estimated empirically with the purpose of avoiding two pit-falls: first that an outcome that is critical to one group might wrongly be excluded due to it being scored low by one or more other groups; and second that the criteria for consensus were too stringent for a significant number of outcomes, leading to excessive weight being placed on the consensus meeting where personal dynamics and stakeholder power may have disproportionate influence.

These issues also hint at the fundamental question of whether the views of all stakeholders are equal in the COS process, and if not, then how should this be weighted. One of the advantages of the COS is that it is not limited to a predetermined small number of outcomes – there is room for the most important outcomes from any and all key stakeholder groups, provided that consensus can be reached.

Identification of the core outcomes for inclusion in the COS is an important step, which will improve data synthesis and allow cross-study comparison. The next phase is to go on to identify and agree measures for consistent assessment of these core outcomes. Candidate measures for these outcomes will have been identified during this project. Future work will include the assessment of the suitability of specific measurement tools aligned to the COS based on their measurement properties including their construct validity, reliability and responsiveness.

Whilst acknowledging some of the challenges to COS development, it is clear that COS has huge potential to improve the value of clinical trials to society and to reduce research waste. The current approach to measuring outcomes in uveitis is acknowledged to be inadequate. The development of a COS for PSIU would be a major step forward for the uveitis community as we seek to improve the treatment of patients with the most sight-threatening uveitis.

### Trial status

At the time of manuscript submission, the status of the trial is ongoing. Patient recruitment has not been completed.

## Abbreviations

COMET: Core Outcome Measures in Effectiveness Trials
COS: Core outcome set
IONA: International Ophthalmic Nurses Associations
OCT: Optical Coherence Tomography
PSIU: Posterior segment Involving uveitis
UMO: Uveitic macular oedema

## References

[1] Durrani O, Meads C, Murray P (2004). Uveitis: a potentially blinding disease. Ophthalmologica.

[2] Williams GJ, Brannan S, Forrester JV, Gavin MP, Paterson-Brown SP, Purdie A (2007). The prevalence of sight-threatening uveitis in Scotland. Br J Ophthalmol.

[3] Gritz DC, Wong IG (2004). Incidence and prevalence of uveitis in Northern California: the Northern California epidemiology of uveitis study. Ophthalmology.

[4] Suhler EB, Lloyd MJ, Choi D, Rosenbaum JT, Austin DF (2008). Incidence and prevalence of uveitis in Veterans Affairs Medical Centers of the Pacific Northwest. Am J Ophthalmol.

[5] Abdulaal MR, Abiad BH, Hamam RN (2015). Uveitis in the aging eye: incidence, patterns, and differential diagnosis. J Ophthalmol.

[6] Rao NA (2013). Uveitis in developing countries. Indian J Ophthalmol.

[7] Vadot E, Barth E, Billet P. Epidemiology of uveitis—preliminary results of a prospective study in Savoy. Uveitis update. Amsterdam: Elsevier; 1984. p. 136.

[8] Al-Dhibi HA, Al-Mahmood AM, Arevalo JF (2014). A systematic approach to emergencies in uveitis. Middle East Afr J Ophthalmol.

[9] de Smet MD, Taylor SR, Bodaghi B, Miserocchi E, Murray PI, Pleyer U (2011). Understanding uveitis: the impact of research on visual outcomes. Prog Retin Eye Res.

[10] Bloch-Michel E, Nussenblatt RB (1987). International Uveitis Study Group recommendations for the evaluation of intraocular inflammatory disease. Am J Ophthalmol.

[11] Jabs DA, Nussenblatt RB, Rosenbaum JT (2005). Standardization of uveitis nomenclature for reporting clinical data. Results of the First International Workshop. Am J Ophthalmol.

[12] Lardenoye CW, van Kooij B, Rothova A (2006). Impact of macular edema on visual acuity in uveitis. Ophthalmology.

[13] Davis J (2010). Current concepts in the management of uveitic macular edema. Adv Stud Ophthalmol.

[14] Jones N (2015). The Manchester Uveitis Clinic: the first 3000 patients, 2: uveitis manifestations, complications, medical and surgical management. Ocul Immunol Inflamm.

[15] Barry RJ, Denniston AK (2015). Controversies in the pharmacological treatment of uveitis. Curr Pharm Des.

[16] Sreekantam S, Denniston AK, Murray PI (2011). Survey of expert practice and perceptions of the supporting clinical evidence for the management of uveitis-related cataract and cystoid macular oedema. Ocul Immunol Inflamm.

[17] Denniston AK, Holland GN, Kidess A, Nussenblatt RB, Okada AA, Rosenbaum JT (2015). Heterogeneity of primary outcome measures used in clinical trials of treatments for intermediate, posterior, and panuveitis. Orphanet J Rare Dis.

[18] Comet Initiative. 2011. http://www.comet-initiative.org/. Accessed 17 Mar 2017.

[19] Williamson PR, Altman DG, Blazeby JM, Clarke M, Devane D, Gargon E (2012). Developing core outcome sets for clinical trials: issues to consider. Trials.

[20] Kirkham JJ, Boers M, Tugwell P, Clarke M, Williamson PR (2013). Outcome measures in rheumatoid arthritis randomised trials over the last 50 years. Trials.

[21] Ward-Smith P (2014). Developing core outcome sets for clinical trials: issues to consider. Urol Nurs.

[22] Hsu C-C, Sandford BA (2007). The Delphi technique: making sense of consensus. Pract Assess Res Eval.

[23] Tallouzi MO, Moore DJ, Calvert M, Murray PI, Bucknall N, Denniston AK (2016). The effectiveness of pharmacological agents for the treatment of uveitic macular oedema (UMO): a systematic review protocol. Syst Rev.

[24] Wilkinson S (1999). Focus groups a feminist method. Psychol Women Q.

[25] Kerr C, Nixon A, Wild D (2010). Assessing and demonstrating data saturation in qualitative inquiry supporting patient-reported outcomes research. Expert Rev Pharmacoecon Outcomes Res.

[26] Carr EC, Worth A (2001). The use of the telephone interview for research. Nurs Times Res.

[27] Novick G (2008). Is there a bias against telephone interviews in qualitative research?. Res Nurs Health.

[28] Braun V, Clarke V. Using thematic analysis in psychology. Qualitative research in psychology. 2006;3(2):77-101.

[29] Sinha IP, Smyth RL, Williamson PR (2011). Using the Delphi technique to determine which outcomes to measure in clinical trials: recommendations for the future based on a systematic review of existing studies. PLoS Med.

[30] Taylor WJ, Schumacher HR, Baraf HS, Chapman P, Stamp L, Doherty M (2008). A modified Delphi exercise to determine the extent of consensus with OMERACT outcome domains for studies of acute and chronic gout. Ann Rheum Dis.

[31] Khanna D, Lovell DJ, Giannini E, Clements PJ, Merkel PA, Seibold JR (2008). Development of a provisional core set of response measures for clinical trials of systemic sclerosis. Ann Rheum Dis.

[32] Payne K, Nicholls SG, McAllister M, MacLeod R, Ellis I, Donnai D (2007). Outcome measures for clinical genetics services: a comparison of genetics healthcare professionals and patients’ views. Health Policy.

[33] Bae JH, Lee CS, Lee SC (2011). Efficacy and safety of intravitreal bevacizumab compared with intravitreal and posterior sub-tenon triamcinolone acetonide for treatment of uveitic cystoid macular edema. Retina.

[34] Hewlett SA (2003). Patients and clinicians have different perspectives on outcomes in arthritis. J Rheumatol.

[35] Clarke M (2007). Standardising outcomes for clinical trials and systematic reviews. Trials.

[36] Rodrigues IA, Sprinkhuizen SM, Barthelmes D, Blumenkranz M, Cheung G, Haller J (2016). Defining a minimum set of standardized patient-centered outcome measures for macular degeneration. Am J Ophthalmol.

[37] Potter S, Holcombe C, Ward JA, Blazeby JM (2015). Development of a core outcome set for research and audit studies in reconstructive breast surgery. Br J Surg.

[38] Potter S, Mills N, Cawthorn S, Wilson S, Blazeby J (2013). Exploring inequalities in access to care and the provision of choice to women seeking breast reconstruction surgery: a qualitative study. Br J Cancer.

[39] Potter S, Mills N, Cawthorn SJ, Donovan J, Blazeby JM (2014). Time to be BRAVE: is educating surgeons the key to unlocking the potential of randomised clinical trials in surgery? A qualitative study. Trials.

[40] Sanderson T, Morris M, Calnan M, Richards P, Hewlett S (2010). What outcomes from pharmacologic treatments are important to people with rheumatoid arthritis? Creating the basis of a patient core set. Arthritis Care Res.

[41] Sanderson T, Morris M, Calnan M, Richards P, Hewlett S (2010). Patient perspective of measuring treatment efficacy: the rheumatoid arthritis patient priorities for pharmacologic interventions outcomes. Arthritis Care Res.

